# Inflammatory Factors of Macular Atrophy in Eyes With Neovascular Age-Related Macular Degeneration Treated With Aflibercept

**DOI:** 10.3389/fimmu.2021.738521

**Published:** 2021-10-13

**Authors:** Tomohito Sato, Toshio Enoki, Yoko Karasawa, Hideaki Someya, Manzo Taguchi, Kozo Harimoto, Kei Takayama, Takayuki Kanda, Masataka Ito, Masaru Takeuchi

**Affiliations:** ^1^ Department of Ophthalmology, National Defense Medical College, Tokorozawa, Japan; ^2^ Enoki Eye Clinic, Sayama, Japan; ^3^ Department of Developmental Anatomy and Regenerative Biology, National Defense Medical College, Tokorozawa, Japan

**Keywords:** aflibercept, aqueous humor cytokine, interferon-γ-inducible protein 10, macrophage inflammatory protein-1β, macular atrophy, monocyte chemoattractant protein-1, neovascular age-related macular degeneration, vascular endothelial growth factor

## Abstract

**Background:**

Neovascular age-related macular degeneration (nAMD) is a leading cause of blindness in older people. Low-grade inflammation is well-known as one of the pathogenic mechanisms in nAMD. Anti-vascular endothelial growth factor (VEGF) therapy is the first-line treatment for nAMD, although macula atrophy (MA) developed under anti-VEGF therapy causes irreversible visual function impairment and is recognized as a serious disorder. Here, we show specific expression patterns of aqueous humor (AH) cytokines in nAMD eyes developing MA under intravitreal injection of aflibercept (IVA) as an anti-VEGF antibody and present predictive cytokines as biomarkers for the incidence of MA in nAMD eyes under IVA treatment.

**Methods:**

Twenty-eight nAMD patients received three consecutive monthly IVA, followed by a *pro re nata* regimen for 2 years. AH specimens were collected before first IVA (pre-IVA) and before third IVA (post-IVA). AH cytokine levels, visual acuity (VA), and central retinal thickness (CRT) were measured.

**Results:**

Two-year incidence of MA was 21.4%. In nAMD eyes developing MA [MA (+) group], pre-IVA levels of monocyte chemoattractant protein-1 (MCP-1), macrophage inflammatory protein (MIP)-1β, VEGF and post-IVA level of MCP-1 were higher than those in nAMD eyes without MA [MA (−) group]. In hierarchical cluster analysis, pre-IVA MCP-1 and VEGF were grouped into the same subcluster, as were post-IVA MCP-1 and CRT. In principal component analysis, principal component loading (PCL) of pre-IVA interferon-γ-inducible protein 10 (IP-10) was 0.61, but PCL of post-IVA IP-10 decreased to −0.09. In receiver operating characteristic analysis and Kaplan–Meier curves, pre-IVA MCP-1, MIP-1β, and VEGF and post-IVA interleukin-6, MCP-1, and MIP-1β were detected as predictive factors for MA incidence. In 2-year clinical course, changes of VA in groups with high levels of pre-IVA MIP-1β (over 39.9 pg/ml) and VEGF (over 150.4 pg/ml) were comparable to those in MA (+) group.

**Conclusion:**

Substantial loss of IP-10 effects and persistent inflammation contribute to incidence of MA, and screening of AH cytokine levels could be a useful method to predict MA incidence in nAMD eyes under anti-VEGF therapy.

## Introduction

In developed countries, age-related macular degeneration (AMD) is a severe ocular disease in people older than 50 years (1). Globally, AMD leads to approximately 1% of visual impairment and some 5% of blindness (2). AMD is classified into two types depending on clinical features: 1) dry type characterized by slow progressive dysfunction of the retinal pigment epithelium (RPE), photoreceptor loss, and retinal degeneration; and 2) neovascular type, which is less frequent but responsible for 90% of acute blindness due to AMD (3). Neovascular AMD (nAMD) is associated with choroidal neovascularization (CNV), leading to subretinal and intraretinal macular edema, hemorrhage, and fibrosis, resulting in visual impairment (4). Vascular endothelial growth factor (VEGF) is the principal mediator of angiogenesis and vascular permeability and contributes to the development of CNV (5). Currently, intravitreal injection of anti-VEGF agent is the first-line therapy for nAMD, and pegaptanib (6), ranibizumab (7), aflibercept (8), and brolucizumab (9), as well as bevacizumab (10), currently not approved for nAMD in Japan, are some of the promising treatments for nAMD.

Recently, macular atrophy (MA) that develops before and/or after anti-VEGF therapy in nAMD eyes has been a concern because MA may cause impairment of visual acuity (VA) and central visual field (11). *Post-hoc* re-gradings of The Comparison of Age-Related Macular Degeneration Treatments Trials (CATT) Study (12), The phase III, double-masked, multicenter, randomized, active treatment-controlled study of the efficacy and safety of 0.5 mg and 2.0 mg ranibizumab administered monthly or on an as-needed basis (PRN) in patients with subfoveal neovascular age-related macular degeneration (HARBOR) Study (11), and The Inhibition of VEGF in Age-related choroidal Neovascularization (IVAN) Study (13) showed that the incidence rates of MA in nAMD eyes under ranibizumab treatment for 2 years were 18.3%, 29.4%, and 29.7%, respectively. Besides, the Development of macular atrophy in patients with neovascular age-related macular degeneration: A comparison of ranibizumab and aflibercept (RIVAL) Study (14) reported 30% incidence rate of MA in nAMD eyes under ranibizumab treatment and 26% under aflibercept treatment for 2 years. However, the etiology of MA remains unclear (11–13), and there is no effective treatment for MA (11).

Accumulated evidence supports low-grade inflammation as one of the pathogenic mechanisms in AMD (5, 15–18). Especially, interferon γ-inducible protein 10 (IP-10), monocyte chemoattractant protein-1 (MCP-1), and VEGF were considered to be involved in the pathophysiology of nAMD (5, 17–19). However, cytokines demonstrate functional multiplicity and diversity by interacting with one another (17, 18, 20, 21). Cytokines secreted by various immune cells express differential responses to target cells (21, 22). Therefore, a comprehensive assessment of cytokines is needed to clarify their functions and influences on the etiology of AMD (18).

The purpose of this study was to identify the specific expression patterns of aqueous humor (AH) cytokines in nAMD eyes that developed MA under treatment with intravitreal injection of aflibercept (IVA) and to detect significant predictors of MA incidence in nAMD eyes.

## Materials and Methods

### Subjects

The studies involving human participants were reviewed and approved by the Ethics Committee of National Defense Medical College, and the procedures conformed to the tenets of the Declaration of Helsinki. The patients/participants provided their written informed consent to participate in this study.

This prospective observational study was performed at the National Defense Medical College Hospital and Enoki Eye Clinic in Japan. The study period was from September 1, 2013, to August 1, 2018, and a consecutive series of 28 treatment-naive eyes in 28 nAMD patients and 29 eyes of 29 cataract patients (controls) was enrolled. Inclusion criteria for the nAMD patients were as follows (17, 18): 1) treatment-naive nAMD eyes with CNV; 2) presence of intraretinal fluid (IRF), subretinal fluid (SRF), or pigment epithelial detachment by spectral-domain optical coherence tomography (SD-OCT); 3) absence of concurrent ocular diseases in the affected eye; 4) refractive power less than −6 diopter and axial length shorter than 26 mm; 5) no history of intraocular surgery other than cataract surgery performed within 6 months before the date of enrollment; and 6) patients who could be followed for 2 years after initiation of IVA. Inclusion criteria for control patients were as follows (17, 18): 1) no current or past history of intraocular inflammatory diseases including AMD, ocular trauma, diabetic retinopathy, retinal artery occlusion, retinal vein occlusion, ocular tumor, and uveitis; 2) refractive power less than −6 diopter and axial length shorter than 26 mm; and 3) no history of intraocular and extraocular surgery. There was no overlap of nAMD patients and controls.

**Figure 1:**
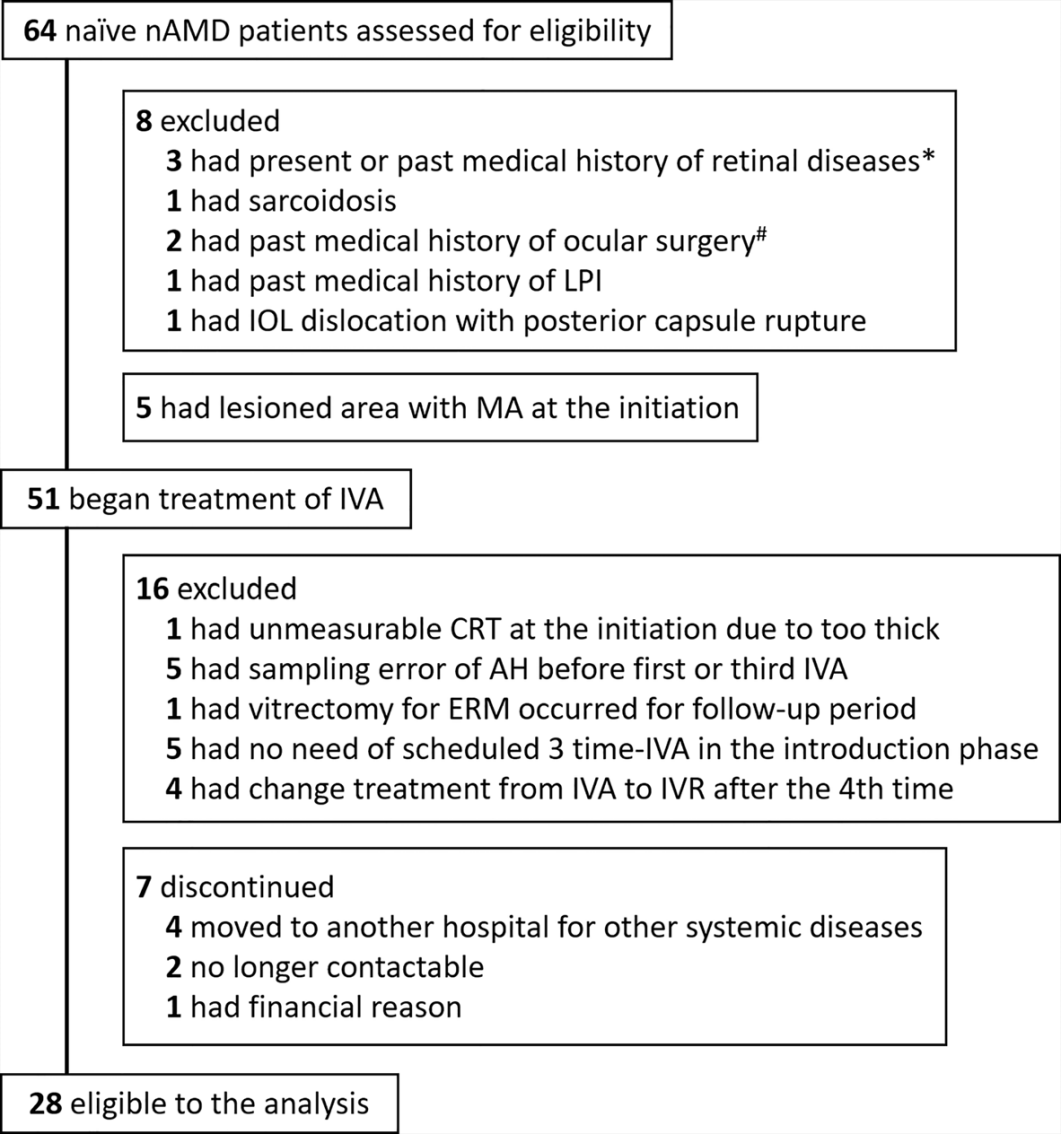
Trial profile. Flowchart of treatment-naive nAMD patients enrolled for IVA treatment is shown. Finally, 28 patients were eligible for analysis in this study. *One patient with branch retinal vein occlusion, one with central serous chorioretinopathy, and one with diabetic macular edema. ^#^One had vitrectomy for epiretinal membrane, and one had scleral buckling for rhegmatogenous retinal detachment. AH, aqueous humor; CRT, central retinal thickness; IOL, intraocular lens; IVA, intravitreal injection of aflibercept; LPI, laser peripheral iridotomy; nAMD, neovascular age-related macular degeneration.

The enrollment process and disposition of nAMD patients are shown in **Figure 1**. 1) At baseline visit, 64 treatment-naive eyes of 64 nAMD patients were enrolled. Thirteen patients were excluded because they were not eligible according to inclusion criteria. 2) Fifty-one patients began IVA treatment. In the observation period, 16 patients were excluded for the following reasons: central retinal thickness (CRT) unmeasurable due to excessive retinal thickening; AH sampling error; intraocular surgery conducted during the observation period; did not complete three consecutive monthly IVA because of disappearance of retinal hemorrhage, IRF, and SRF; switch from aflibercept to other anti-VEGF agents such as ranibizumab; and discontinuation of hospital visits. 3) Finally, 28 nAMD eyes under treatment with IVA were evaluated.

All the 28 nAMD eyes analyzed received three consecutive monthly treatments (weeks 0, 4, and 8) of 2 mg aflibercept by intravitreal injection according to the protocol reported previously (17). Thereafter, IVA was given following a *pro re nata* (*PRN*) regimen using findings of color fundus photography and/or SD-OCT images to guide subsequent injection period (23, 24). In the *PRN* regimen, IVA was scheduled according to the criteria of disease activity as follows: 1) loss of (0.2) decimal VA or more compared to the best-corrected visual acuity (BCVA) recorded after the start of aflibercept treatment; or 2) recurrence and exacerbation of retinal hemorrhage, IRF, or SRF.

In the present study, we performed *a priori* power calculation using our previous study data (17, 18). To confirm significant differences in AH cytokine levels with a statistical power of 0.80 (25), the sample size of the study and control arms should be approximately 20 each. Therefore, we attempted to recruit 20 or more cases in total nAMD group and control group in this study.

### Diagnostics and Treatments

Diagnosis of nAMD was based on a full ophthalmological examination including BCVA test using a decimal chart, intraocular pressure measurement, slit-lamp biomicroscopy, dilated fundus examination, color fundus photography, fundus fluorescein and indocyanine green angiography, and SD-OCT (Cirrus HD-OCT; Carl Zeiss Meditec, Dublin, CA, USA). The subtypes of nAMD were classified into Type I CNV, Type II CNV, polypoidal choroidal vasculopathy (PCV), and retinal angiomatous proliferation (RAP) according to the classification and diagnostic criteria of AMD (26, 27). BCVA was converted to logarithm of the minimum angle of resolution (logMAR) units (logMAR VA) for statistical analysis. Counting fingers, hand motion, light perception (+), and light perception (−) were converted to 1.85 logMAR, 2.30 logMAR, 2.80 logMAR, and 2.90 logMAR, respectively, in accordance with previous reports (28, 29). CRT was defined as the mean retinal thicknesses of a central 1-mm circle on the Early Treatment Diabetic Retinopathy Study grid (30) in the macula (17, 18) and measured by the SD-OCT. MA in nAMD eye was diagnosed according to the definition in the HARBOR Study (11). In brief, the diagnostic criteria of MA were as follows: 1) sharply demarcated areas of RPE depigmentation on color fundus photography or fluorescein angiography; 2) circular borders or irregular in shape with straight edges or margins; and 3) diameter of atrophic area ≥250 mm.

Three retinal specialists, who are members of Japanese Retina and Vitreous Society, diagnosed and reviewed the presence of MA in the subjects. In case of discrepancy among the three assessors, the decision was adjudicated by majority rule.

### Aqueous Humor Sample Collection and Cytokine Measurements

AH samples from nAMD patients were collected before the first IVA (pre-IVA) and before the third IVA (post-IVA). At each sampling, approximately 0.1 ml of undiluted AH was collected by performing an anterior chamber limbal paracentesis. In controls, undiluted AH samples were obtained at the beginning of cataract surgery. No complication associated with sampling of AH occurred. The AH samples were stored at −80°C until processing. Twenty-seven types of inflammatory cytokines expected to provide a comprehensive coverage of inflammatory mediators (Bio-Plex Human Cytokine 27-plex panel; Bio-Rad, Hercules, CA, USA) were measured by a multiplex bead analysis system (Bio-Plex Suspension Array System; Bio-Rad) according to manufacturers’ instructions. All standards and samples were assayed in duplicate. Levels of AH cytokines below detectable levels were treated as 0 for statistical analysis (5, 17, 18).

### Statistical Analysis

Statistical analyses were performed using the statistic add-in software for Excel (BellCurve for Excel^®^, SSRI Co., Ltd., Tokyo, Japan, and XLSTAT^®^, Addinsoft Company, Paris, France). Data are expressed as mean ± standard deviation (17). Mann–Whitney U test and Spearman’s rank correlation test were used for nonparametric comparison of unpaired two groups. Wilcoxon signed-rank test was used to compare paired two groups. Kruskal–Wallis test followed by *post-hoc* Steel–Dwass test was used for nonparametric comparison of unpaired multiple groups. Fisher’s exact test (for n < 4) was used to compare categorical variables. Inflammatory cytokines with over 50% of detection rate were adopted as explanatory variables in hierarchical cluster analysis, principal component analysis (PCA), and receiver operating characteristic (ROC) curve analysis. Hierarchical cluster analysis was performed using Euclidean distance as a distance measure and Ward’s method for hierarchical clustering (18). Kaplan–Meier curves were compared using Cochran–Mantel–Haenszel log-rank test. Two-tailed test was applied in all of the statistical methods. A p-value <0.05 was considered to be statistically significant.

## Results

### Subjects

The disposition of nAMD patients enrolled in this study is summarized in **Figure 1**. Initially, 64 treatment-naive nAMD patients were assessed for eligibility according to our inclusion criteria, and finally 28 treatment-naive nAMD eyes of 28 patients (43.8%) were analyzed. Twenty-nine cataract eyes of 29 patients were included as the control group. The demographic and clinical characteristics in the nAMD patients (total nAMD group) are shown in **Supplementary Table S1**. Total nAMD group was composed of 10 eyes (35.7%) classified as typical AMD subdivided into type I CNV (Type I: five eyes, 17.9%) and type II CNV (Type II: five eyes, 17.9%), 18 eyes (64.3%) as PCV, and 0 eye as RAP. The proportion of males in total nAMD group was higher than that in control group, although age was not significantly different between the two groups. The levels of AH cytokines in total nAMD group before the first IVA and before the third IVA are shown in **Supplementary Table S2**.

The clinical characteristics of nAMD eyes developing MA during 2 years of IVA treatment [MA (+) group: six eyes of six patients, 21.4%] and nAMD eyes without MA [MA (−) group: 22 eyes of 22 patients, 78.6%] are summarized in **Table 1**. MA (+) group was composed of one eye (16.7%) with Type I, two eyes (33.3%) with Type II, and three eyes (50.0%) with PCV. MA (−) group consisted of four eyes (18.2%) with Type I, three eyes (13.6%) with Type II, and 15 eyes (68.2%) with PCV. There were no significant differences in age, gender, and nAMD subtypes between the two groups (**Table 1A**). The numbers of IVA doses received in 2 years in MA (+) group and MA (−) group are shown in **Table 1B**. The numbers of IVA doses did not differ significantly between the two groups in all the indicated periods. Pre-IVA VA and post-IVA VA (logMAR) were significantly higher (p = 0.017 and p = 0.008, respectively) in MA (+) group than those in MA (−) group, although pre-IVA CRT and post-IVA CRT did not differ significantly between the two groups (**Table 2**).

**Table 1:** Clinical characteristics and number of aflibercept treatment for 2 years in nAMD patients with or without incidence of macular atrophy under aflibercept treatment.

A				B			
Category	MA (+)	MA (−)	p value	nAMD	MA (+)	MA (−)	p value
*n*	6	22		Category	Mean ± SD		
Age (year)	77.0^#^ (70 - 85)^†^	67.5 (43 - 83)	0.067
Gender (M / F)	18 / 4	3 / 3	1.000	**0 to 2 mo.**	3.00	2.86 ± 0.35	0.520
Subtype				**3 to 6 mo.**	1.17 ± 0.41	1.05 ± 0.72	0.524
Typical	3 (50.0%)	7 (31.8%)	0.634^a^	**6 to 12 mo.**	3.00 ± 1.26	2.41 ± 1.26	0.393
Type I	1 (16.7%)	4 (18.2%)	0.570^b^	**0 to 12 mo.**	7.17 ± 1.60	6.32 ± 1.59	0.380
Type II	2 (33.3%)	3 (13.6%)		**12 to 24 mo.**	5.00 ± 3.58	4.73 ± 2.78	0.562
PCV	3 (50.0%)	15 (68.2%)	** **	**0 to 24 mo.**	12.2 ± 5.04	11.1 ± 4.04	0.533

(**A**) Clinical characteristics and (**B**) number of IVA doses for 2 years in nAMD patients with or without incidence of MA under aflibercept treatment are shown. F, female; IVA, intravitreal injection of aflibercept; M, male; MA, macular atrophy; MA (+), nAMD eyes developing MA; MA (−), nAMD eyes without MA; mo., month; n, number; nAMD, neovascular age-related macular degeneration; PCV, polypoidal choroidal vasculopathy; SD, standard deviation.

a typical vs. PCV.

b among Type 1, Type 2, and PCV; ^#^average; ^†^range.

**Table 2:** Visual acuity, central retinal thickness, and aqueous humor cytokine levels in nAMD patients with or without incidence of macular atrophy under aflibercept treatment.

Category	Pre-IVA				Post-IVA				*p* value			
Subgroup	MA (＋)		MA (－)		MA (＋)		MA (－)		Pre-IVA	Post-IVA	MA (＋)	MA (－)
*n*	6		22		6		22		MA (＋) *vs.* MA (－)	MA (＋) *vs.* MA (－)	Pre-IVA *vs.* Post-IVA	Pre-IVA *vs.* Post-IVA
	Detectable rate (%)	Mean ± SD	Detectable rate (%)	Mean ± SD	Detectable rate (%)	Mean ± SD	Detectable rate (%)	Mean ± SD				
**logMAR VA**	6 (100)	0.96 ± 0.67	22 (100)	0.26 ± 0.28	6 (100)	0.95 ± 0.63	22 (100)	0.16 ± 0.33	**0.017**	**0.008**	1.000	**0.027**
**CRT**	6 (100)	377.2 ± 86.5	22 (100)	307.6 ± 64.6	6 (100)	252.0 ± 62.3	22 (100)	231.1 ± 38.2	0.084	0.352	**0.047**	**4.64 x 10^-5^**
PDGF-BB	0 (0)	0	0 (0)	0	0 (0)	0	0 (0)	0	–	–	–	–
IL-1β	0 (0)	0	2 (9.09)	0 ± 0.01	0 (0)	0	4 (18.2)	0	0.540	0.501	–	1.000
IL-1ra	0 (0)	0	0 (0)	0	0 (0)	0	2 (9.09)	1.27 ± 4.13	–	0.540	–	0.183
IL-2	0 (0)	0	0 (0)	0	0 (0)	0	0 (0)	0	–	–	–	–
IL-4	0 (0)	0	4 (18.2)	0.03 ± 0.07	0 (0)	0	2 (9.09)	0.02 ± 0.05	0.501	0.540	–	0.363
IL-5	0 (0)	0	0 (0)	0	0 (0)	0	0 (0)	0	–	–	–	–
**IL-6**	3 (50.0)	6.17 ± 7.59	15 (68.2)	4.59 ± 8.52	6 (100)	16.1 ± 17.9	15 (68.2)	7.88 ± 11.9	0.549	0.067	**0.028**	**0.049**
IL-7	6 (100)	9.74 ± 4.98	22 (100)	7.15 ± 5.39	6 (100)	11.7 ± 9.99	20 (90.9)	8.42 ± 6.99	0.163	0.286	0.611	0.326
**IL-8**	4 (66.7)	9.40 ± 9.70	11 (50.0)	2.56 ± 4.73	5 (83.3)	11.7 ± 12.0	16 (72.7)	8.17 ± 11.1	0.149	0.324	0.500	**0.001**
IL-9	1 (16.7)	0.35 ± 0.86	0 (0)	0	1 (16.7)	0.62 ± 1.51	0 (0)	0	0.508	0.508	0.668	–
IL-10	0 (0)	0	0 (0)	0	0 (0)	0	0 (0)	0	–	–	–	–
**IL-12**	4 (66.7)	8.72 ± 9.03	10 (45.5)	5.77 ± 8.01	0 (0)	0	0 (0)	0	0.380	–	0.068	**0.005**
IL-13	3 (50.0)	1.90 ± 2.92	9 (40.9)	1.20 ± 1.74	2 (33.3)	1.96 ± 4.05	7 (31.8)	1.17 ± 2.43	0.530	0.572	1.000	0.802
IL-15	0 (0)	0	0 (0)	0	0 (0)	0	0 (0)	0	–	–	–	–
IL-17A	0 (0)	0	3 (13.6)	0.03 ± 0.09	0 (0)	0	3 (13.6)	0.13 ± 0.40	0.520	0.520	–	0.151
Eotaxin	3 (50.0)	3.09 ± 3.49	13 (59.1)	2.25 ± 3.49	3 (50.0)	4.41 ± 5.04	14 (63.6)	3.22 ± 3.76	0.536	0.546	0.467	0.248
bFGF	0 (0)	0	7 (31.8)	0.66 ± 1.10	1 (16.7)	2.67 ± 6.54	7 (31.8)	0.69 ± 1.24	0.253	0.536	0.319	1.000
G-CSF	1 (16.7)	0.97 ± 2.37	0 (0)	0	0 (0)	0	0 (0)	0	0.508	–	0.319	–
GM-CSF	0 (0)	0	0 (0)	0	0 (0)	0	0 (0)	0	–	–	–	–
IFN-γ	1 (16.7)	1.12 ± 2.74	0 (0)	0	0 (0)	0	0 (0)	0	0.508	–	0.319	–
**IP-10**	6 (100)	858.2 ± 946.5	21 (95.5)	492.5 ± 467.5	6 (100)	2548.1 ± 4750.8	22 (100)	1456.2 ± 1568.6	0.380	0.520	0.178	**1.82 x 10^-4^**
**MCP-1**	6 (100)	280.4 ± 192.2	22 (100)	137.6 ± 141.8	6 (100)	248.2 ± 149.6	22 (100)	155.6 ± 164.1	**0.018**	**0.048**	0.922	**0.037**
**MIP-1α**	5 (83.3)	2.44 ± 2.73	7 (31.8)	0.10 ± 0.19	3 (50.0)	1.15 ± 1.66	10 (45.5)	0.45 ± 0.81	**0.007**	0.441	0.611	0.091
**MIP-1β**	6 (100)	46.4 ± 25.3	22 (100)	21.8 ± 22.3	6 (100)	38.7 ± 19.1	22 (100)	27.1 ± 29.8	**0.029**	0.106	0.922	0.088
RANTES	0 (0)	0	3 (13.6)	0.06 ± 0.16	0 (0)	0	2 (9.09)	0.20 ± 0.71	0.520	–	–	0.467
TNFα	0 (0)	0	0 (0)	0	0 (0)	0	0 (0)	0	–	0.540	–	–
**VEGF**	6 (100)	269.3 ± 293.2	15 (68.2)	70.1 ± 66.8	0 (0)	0	1 (4.55)	0.42 ± 1.97	**0.026**	0.559	**0.028**	**0.001**

LogMAR VA, CRT, and AH cytokine levels in nAMD patients with or without MA incidence under IVA treatment for 2 years are shown. AH, aqueous humor; bFGF, basic fibroblast growth factor; CRT, central retinal thickness; G-CSF, granulocyte colony-stimulating factor; GM-CSF, granulocyte-macrophage colony-stimulating factor; PDGF-BB, platelet-derived growth factor-BB; IL, interleukin; IFN-γ, interferon-gamma; IP-10, interferon γ-inducible protein 10; IVA, intravitreal injection of aflibercept; pre-IVA, before first IVA; post-IVA, before the third IVA; logMAR, logarithm of the minimum angle of resolution; MCP-1, monocyte chemotactic protein-1; MIP, macrophage inflammatory protein; ra, receptor antagonist; RANTES, regulated on activation, normal T cell expressed and secreted; TNFα, tumor necrosis factor α; VA, visual acuity; VEGF, vascular endothelial growth factor. Examination items with significant differences and its p values are shown in bold.

Pre-IVA VA was significantly higher (p = 0.027) than post-IVA VA in MA (−) group but did not differ in MA (+) group. Pre-IVA CRT was significantly higher than post-IVA CRT in both MA (+) and MA (−) groups (p = 0.047 and p = 4.64 × 10^-5^, respectively).

### Aqueous Humor Cytokine Levels Before and After Initiation of Aflibercept in nAMD Eyes

The profiles of AH cytokine levels in total nAMD group and control group are summarized in **Supplementary Table S2**. Pre-IVA IP-10 level in total nAMD group was higher and interleukin (IL)-1 receptor antagonist (ra), IL-6, and interferon (IFN)-γ levels were lower compared to the levels in control group. Post-IVA eotaxin and IP-10 levels in total nAMD group were higher and IL-1ra, IL-12, IFN-γ, and VEGF levels were lower compared to the levels in control group. In total nAMD group, post-IVA IL-6, IL-8, and IP-10 levels were higher and IL-12 and VEGF levels were lower than the pre-IVA levels of these cytokines.

The profiles of AH cytokine levels in MA (+) group and MA (−) group are summarized in **Table 2**. Pre-IVA MCP-1, macrophage inflammatory protein (MIP)-1α, MIP-1β, and VEGF levels were higher in MA (+) group than those in MA (−) group. Post-IVA MCP-1 level was higher in MA (+) group than that in MA (−) group. In MA (+) group, post-IVA IL-6 level was higher and post-IVA VEGF level was lower than pre-IVA levels of these cytokines. In MA (−) group, post-IVA IL-8, IP-10, and MCP-1 levels were elevated compared to pre-IVA levels of these cytokines, while post-IVA IL-12 level was lower than the pre-IVA level.

### Expression Patterns of Visual Acuity, Central Macula Thickness, and Cytokines of Aqueous Humor by Hierarchical Cluster Analysis in nAMD Eyes With or Without Macular Atrophy

**Figure 2:**
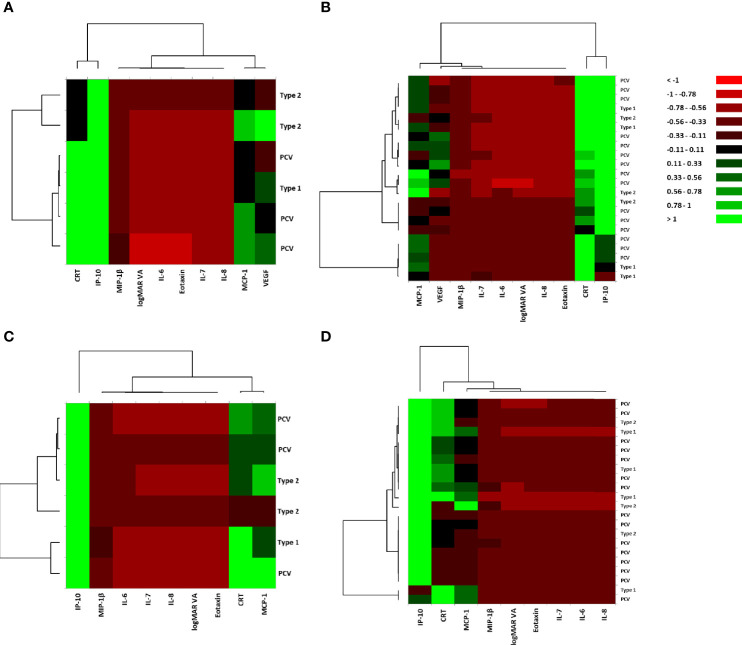
Hierarchical cluster analysis for the variables of visual acuity, central macula thickness, and aqueous humor cytokines in nAMD eyes with or without incidence of macular atrophy under aflibercept treatment. Heat maps of logMAR VA, CRT, and AH cytokines in **(A)** MA (+) group pre-IVA, **(B)** MA (−) group pre-IVA, **(C)** MA (+) group post-IVA, and **(D)** MA (−) group post-IVA are shown. Color scale: low values, red; middle to high values, black to green. Vertical axis shows subtypes of nAMD, and horizontal axis demonstrates logMAR VA, CRT, and aqueous humor cytokines. AH, aqueous humor; CRT, central retinal thickness; IVA, intravitreal injection of aflibercept; MA, macular atrophy; MA (+) group, nAMD eyes developing MA; MA (−) group, nAMD eyes without MA; logMAR, logarithm of the minimum angle of resolution; nAMD, neovascular age-related macular degeneration; VA, visual acuity.

For pre-IVA variables, the explanatory variables were broadly classified into two principal clusters: 1) a cluster composed of CRT and IP-10, and 2) a cluster consisting of logMAR VA and the hierarchically selected cytokines, in both MA (+) group (**Figure 2A**) and MA (−) group (**Figure 2B**). In MA (+) group, MCP-1 was proximately located to VEGF, although it was placed independently in MA (−) group. However, MCP-1 and VEGF were grouped into the same subcluster in MA (+) group but were grouped into different subclusters in MA (−) group.

For post-IVA variables, the explanatory variables were roughly divided into one cytokine and one principal cluster: 1) IP-10 and 2) a cluster consisting of logMAR VA, CRT, and the hierarchically selected cytokines in both MA (+) group (**Figure 2C**) and MA (−) group (**Figure 2D**). In MA (+) group, CRT was proximately located to MCP-1, although it was placed independently in MA (−) group. CRT and MCP-1 were grouped into the same subcluster in MA (+) group, but they were grouped into different subclusters in MA (−) group.

### Expression Patterns of Visual Acuity, Central Macula Thickness, and Cytokines of Aqueous Humor by Principal Component Analysis in nAMD Eyes With or Without Macular Atrophy

**Figure 3:**
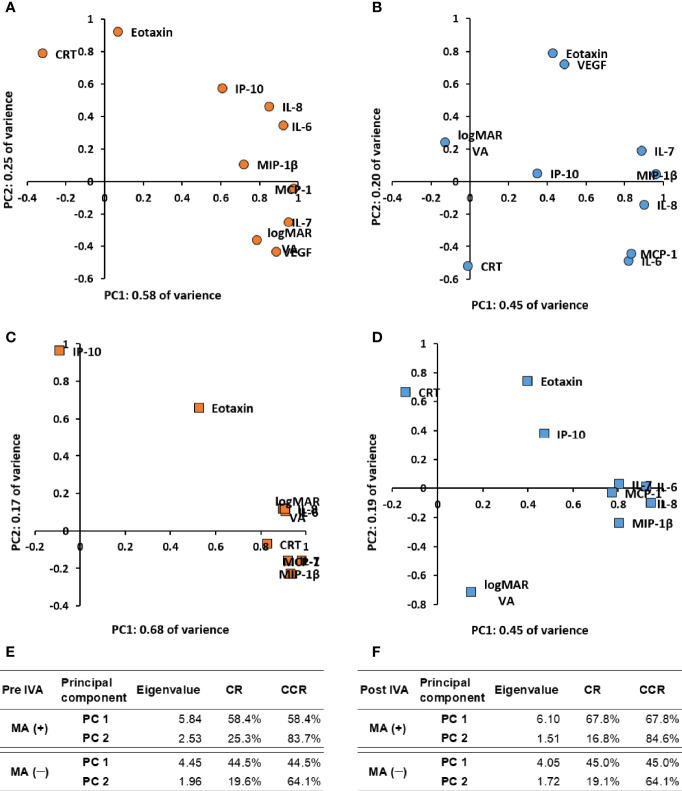
Expression patterns of visual acuity, central macula thickness, and aqueous humor cytokines by principal component analysis in nAMD eyes with or without development of macular atrophy under aflibercept treatment. Biplots of PCLs in PC1 and PC2 of **(A)** MA (+) group pre-IVA, **(B)** MA (−) group pre-IVA, **(C)** MA (+) group post-IVA, and **(D)** MA (−) group post-IVA are shown. Eigenvalues, CR, and CCR of PC1 and PC2 are presented in **(E)** MA (+) group and MA (−) group pre-IVA and **(F)** MA (+) group and MA (−) group post-IVA. CCR, cumulative contribution ratio; CR, contribution ratio; IVA, intravitreal injection of aflibercept; MA, macular atrophy; nAMD, neovascular age-related macular degeneration; PC, principal component; PCL, principal component loading; PC1, first principal component; PC2, second principal component.

Scatter plots of principal component loadings (PCLs) for first principal component (PC1, x-axis) and second PC (PC2, y axis) are shown in **Figure 3**. For pre-IVA variables, PC1 accounted for 58.4% of the variance of the entire dataset in MA (+) group (**Figures 3A, E**). All the cytokines except eotaxin formed a big cluster together with logMAR VA. The PCL of eotaxin was approximately 0.07 and had almost no contribution to PC1. On the other hand, CRT was located on the opposite side of the big cluster in PC1. The PC2 (y-axis) accounted for 25.3% of the variance of the dataset, and CRT was located on the other side of logMAR VA. Based on the dispositions of pre-IVA logMAR VA, -CRT, and -inflammatory cytokines in the scatter plots, the properties of all inflammatory cytokines examined excluding eotaxin were similar to some extent with that of logMAR VA in MA (+) group, and those cytokines would be associated with pre-IVA logMAR VA. On the other hand, the property of pre-IVA CRT had little resemblance to that of pre-IVA logMAR VA and was similar in some degree to that of eotaxin. In MA (−) group, PC1 accounted for 44.5% of the variance of the dataset (**Figures 3B, E**). The cytokines were roughly classified into one independent cytokine and three groups as follows: 1) IP-10; 2) a group of eotaxin and VEGF; 3) a group of IL-6 and MCP-1; and 4) a group composed of IL-7, IL-8, and MIP-1β. LogMAR VA and CRT were located independently from IP-10 and the three cytokine groups. The PCLs of logMAR VA and CRT were –0.13 and –0.01 respectively, and had almost no contribution to the PC1. PC2 accounted for 19.6% of the variance of the dataset. In the PC2, the group of eotaxin and VEGF was located on the opposite side of the group of IL-6 and MCP-1. LogMAR VA was located on the other side of CRT. Based on the arrangements of pre-IVA logMAR VA, -CRT, and -inflammatory cytokines, the properties of all cytokines evaluated were not similar with those of pre-IVA logMAR VA and -CRT in MA (−) group. Furthermore, the property of pre-IVA CRT had little resemblance to that of pre-IVA logMAR VA. On the other hand, the group of eotaxin and VEGF would have conflicting effects to the cluster of IL-6 and MCP-1.

For post-IVA variables, PC1 accounted for 67.8% of the variance of the entire dataset in MA (+) group (**Figures 3C, F**). All the cytokines except eotaxin and IP-10 formed a tight cluster together with logMAR VA and CRT. IP-10 with PCL of approximately −0.09 in PC1 had the weakest contribution to PC1. PC2 accounted for 16.8% of the variance of the dataset. In the PC2, eotaxin was located on the same side of IP-10. These cytokines were located on the opposite side of the group composed of IL-7, MCP-1, and MIP-1β. Based on the disposition of post-IVA logMAR VA, -CRT, and -inflammatory cytokines, the properties of post-IVA logMAR VA, -CRT, and -all cytokines examined excluding eotaxin and IP-10 were similar in MA (+) group. The property of IP-10 was different with that of the big cluster; in other words, IP-10 had little influence to the big cluster. In MA (−) group, PC1 accounted for 45.0% of the variance of the dataset (**Figures 3D, F**). The cytokines were broadly divided into two independent cytokines and one big group: 1) eotaxin; 2) IP-10; and 3) a tight group composed of the hierarchically selected cytokines. LogMAR VA and CRT were located independently from the two cytokines and the big group and had PCLs of 0.15 and −0.14, respectively, in the PC1. Therefore, logMAR VA and CRT had almost no contribution to the PC1. PC2 accounted for 19.1% of the variance of the dataset. In the PC2, eotaxin, IP-10, and CRT were located on the opposite side of logMAR VA. Based on the arrangements of post-IVA logMAR VA, -CRT, and -inflammatory cytokines, the properties of all cytokines evaluated were not similar with those of post-IVA logMAR VA and -CRT, and the property of post-IVA CRT had little resemblance to that of post-IVA logMAR VA in MA (−) group, same as pre-IVA.

### Correlation Between Aqueous Humor Cytokine Levels and Visual Acuity or Central Macula Thickness in nAMD Eyes With or Without Incidence of Macular Atrophy

The correlation between AH cytokine levels and VA or CRT in MA (+) group and MA (−) group was analyzed by Spearman’s rank correlation test. **Table 3** shows the p-values, and **Supplementary Table S3** presents the Spearman’s rank correlation coefficients. For pre-IVA variables, there was no significant correlation between logMAR VA and CRT or any of the cytokine levels in both MA (+) group and MA (−) group. Likewise, CRT did not correlate with any of the cytokine levels. For post-IVA variables, in MA (+) group, logMAR VA correlated significantly with IL-7 (p = 0.020, *r_s_* = 0.883) and with MIP-1β (p = 0.031, *r_s_* = 0.863), while CRT correlated positively with MCP-1 level (p = 0.019, *r_s_* = 0.886). In MA (−) group, there was no significant correlation between the AH cytokine levels and VA or CRT after IVA. Based on the results, pre-IVA logMAR VA and -CRT in the individual nAMD eye whether MA (+) group or MA (−) group were not estimated by pre-IVA levels of IL-6, IL-7, IL-8, eotaxin, IP-10, MCP-1, MIP-1β, and VEGF and may not be associated with those of the cytokines. In the eye of MA (+) group, post-IVA logMAR VA was estimated by post-IVA levels of IL-7 and MIP-1β, and post-IVA CRT was predicted by post-IVA MCP-1 level. In particular, post-IVA levels of MIP-1β and MCP-1 were 38.7 pg/ml and 248.2 pg/ml, respectively, and those levels would be sufficiently high as stably measurable cytokine levels.

**Table 3:** Correlation between aqueous humor cytokine levels and visual acuity or central retinal thickness in nAMD eyes with or without incidence of macular atrophy under aflibercept treatment.

*p* value	Pre IVA									
MA (+)	logMAR VA	CRT	IL-6	IL-7	IL-8	Eotaxin	IP-10	MCP-1	MIP-1β	VEGF
logMAR VA	–	0.872	0.295	0.072	0.499	0.725	0.156	0.266	0.266	0.072
CRT		–	0.686	0.957	0.827	0.055	0.704	0.872	0.468	0.957
IL-6			–	**0.021**	**0.003**	0.322	0.231	**0.021**	0.123	**0.021**
IL-7			*	–	**0.036**	0.816	0.266	**0.005**	**0.042**	**≒ 0**
IL-8			**	*	–	0.423	0.354	**0.008**	0.148	**0.036**
Eotaxin						–	0.908	0.816	0.510	0.816
IP-10							–	0.397	0.957	0.266
MCP-1			*	**	**			–	0.072	**0.005**
MIP-1β				*					–	**0.042**
VEGF			*		*			**	*	–
**MA (−)**	logMAR VA	CRT	IL-6	IL-7	IL-8	Eotaxin	IP-10	MCP-1	MIP-1β	VEGF
logMAR VA	–	0.248	0.324	0.197	0.356	0.910	0.931	0.733	0.913	0.990
CRT		–	0.312	0.642	0.576	0.055	0.631	0.644	0.571	0.567
IL-6			–	**0.007**	**0.026**	0.658	0.053	**0.037**	**0.004**	0.151
IL-7			**	–	**0.037**	0.201	**0.027**	0.072	**7.96 x 10^-5^**	**0.001**
IL-8			*	*	–	0.364	0.519	**0.026**	0.057	0.397
Eotaxin						–	0.924	0.782	0.309	0.177
IP-10				*			–	**0.017**	**0.001**	**0.009**
MCP-1			*		*		*	–	**0.010**	0.276
MIP-1β			**	**			**	*	–	**8.03 x 10^-6^**
VEGF				**			**		**	–
***p* value**	**Post IVA**									
**MA (+)**	logMAR VA	CRT	IL-6	IL-7	IL-8	Eotaxin	IP-10	MCP-1	MIP-1β	
logMAR VA	–	0.824	0.076	**0.020**	0.612	0.546	≒ 1	0.781	**0.031**	
CRT		–	0.872	0.266	0.208	0.123	0.623	**0.019**	0.544	
IL-6			–	0.208	0.156	0.686	0.266	0.872	0.208	
IL-7	*			–	0.468	0.231	0.957	0.329	**0.019**	
IL-8					–	0.364	**0.019**	0.208	0.704	
Eotaxin						–	0.600	0.439	0.774	
IP-10					*		–	0.623	0.704	
MCP-1		*						–	0.468	
MIP-1β	*			*					–	
**MA (−)**	logMAR VA	CRT	IL-6	IL-7	IL-8	Eotaxin	IP-10	MCP-1	MIP-1β	
logMAR VA	–	0.330	0.751	0.452	0.992	0.097	0.302	0.936	0.825	
CRT		–	0.600	0.827	0.977	0.388	0.857	0.962	0.597	
IL-6			–	**0.004**	**0.005**	0.280	0.071	**0.007**	**0.022**	
IL-7			**	–	**0.002**	0.318	0.095	0.071	**2.25 x 10^-4^**	
IL-8			**	**	–	0.379	0.093	**2.83 x 10^-4^**	**0.002**	
Eotaxin						–	0.224	0.139	0.710	
IP-10							–	0.064	0.076	
MCP-1			**		**			–	0.253	
MIP-1β			*	**	**				–	

Abbreviations for items evaluated are as shown in **Tables 1** and **2**. ≒, nearly equal; *p < 0.05; **p < 0.01. P values with less than 0.05 are shown in bold.

In the comparisons within MA (+) or MA (−) group, pre-IVA logMAR VAs correlated positively with post-IVA logMAR VAs in both MA (+) group and MA (−) group (**Supplementary Figures S1, S2**). Moreover, pre-IVA IL-7 and -IL-8 levels showed a positive correlation with post-IVA levels of these cytokines in MA (+) group. In MA (−) group, pre-IVA IL-6, IL-7, IP-10, and MIP-1β levels correlated positively with post-IVA levels of these cytokines. Based on the results, post-IVA logMAR VA in the individual nAMD eye whether MA (+) group or MA (−) group was estimated by pre-IVA logMAR VA, although post-IVA CRT was not predicted by pre-IVA CRT. In the eye of MA (+) group, the levels of IL-6, eotaxin, IP-10, MCP-1, and MIP-1β were irregularly changed by IVA, and the levels of eotaxin and MCP-1 in the eye of MA (−) group were fluctuated without regularity.

### Predictive Factors for Incidence of Macular Atrophy in nAMD Eyes Under Aflibercept Treatment

**Figure 4:**
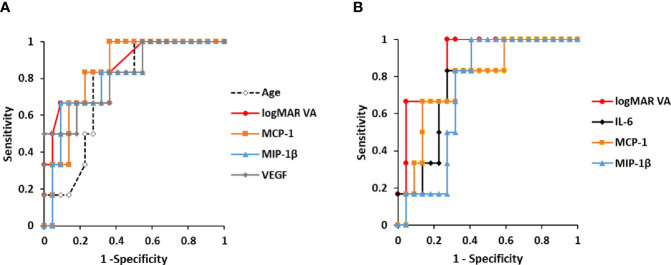
Predictive factors for the incidence of macular atrophy in nAMD eyes under aflibercept treatment. **(A)** ROC curves of age, pre-IVA logMAR VA, and pre-IVA levels of MCP-1, MIP-1β, and VEGF are shown as predictive factors for MA incidence in nAMD eyes under IVA treatment for 2 years. **(B)** ROC curves of post-IVA logMAR VA and post-IVA levels of IL-6, MCP-1, and MIP-1β are presented. Cutoff value is defined by closest point to upper left-hand corner of graph. IL, interleukin; IVA, intravitreal injection of aflibercept; MA, macular atrophy; logMAR, logarithm of the minimum angle of resolution; MCP-1, monocyte chemotactic protein-1; MIP-1β, macrophage inflammatory protein; nAMD, neovascular age-related macular degeneration; ROC, receiver operating characteristic; VEGF, vascular endothelial growth factor.

Age, logMAR VA, CRT, and cytokines with detection rates over 50% (**Table 2**) were considered to be variables with reliable measurement accuracy and were evaluated as candidate predictors of MA incidence by ROC curve analysis. The ROC curves of significant predictors are shown in **Figure 4**, and the detailed results of ROC curve analysis for all the parameters are shown in **Supplementary Table S4**.

For pre-IVA variables, age, logMAR VA, MCP-1, MIP-1β, and VEGF levels were significant predictors of MA incidence (**Figure 4A**). The cutoff values of these factors were over 75 years of age, 0.824, 120.8 pg/ml, 39.9 pg/ml, and 150.4 pg/ml, respectively. For post-IVA variables, logMAR VA, IL-6, MCP-1, and MIP-1β levels significantly predicted MA incidence (**Figure 4B**). The cutoff values of these factors were over 0.301, 7.01 pg/ml, 152.8 pg/ml, and 26.8 pg/ml, respectively.

### Relationship Between Incidence of Macular Atrophy and Age, Visual Acuity, or Aqueous Humor Cytokine Levels in nAMD Eyes Under Aflibercept Treatment

The relationship between MA incidence and the predictive factors detected by the ROC curve analysis (**Figure 4**) was evaluated using Fisher’s exact test (**Table 4**). Total nAMD group was divided into two subgroups: 1) the cutoff value of the predictive factor or more (high-value subgroup) and 2) less than the cutoff value (low-value subgroup).

**Table 4:** Relationship between incidence of macular atrophy and age, visual acuity, or aqueous humor cytokine levels in nAMD eyes under aflibercept treatment.

Category	Cut-off value	High-value subgroup		Low-value subgroup		Cramer's V	Yule's Q	*p* value
*n* = 28		MA (+)	MA (−)	MA (+)	MA (−)			
**Age**	75	5* (83.3)^†^	6	1	16	0.471	0.860	**0.022**
**Pre IVA**								
**LogMAR VA**	0.824	4 (66.7)	2	2	20	0.576	0.905	**0.010**
**MCP-1**	120.8	5 (83.3)	5	1	17	0.519	0.889	**0.013**
**MIP-1β**	39.87	4 (66.7)	2	2	20	0.576	0.905	**0.010**
**VEGF**	150.4	4 (66.7)	4	2	18	0.440	0.800	**0.038**
**Post IVA**								
**LogMAR VA**	0.301	6 (100)	6	0	16	0.603	1.000	**0.002**
**IL-6**	7.010	5 (83.3)	6	1	16	0.471	0.860	**0.022**
MCP-1	152.8	5 (83.3)	7	1	15	0.427	0.829	0.057
MIP-1β	26.84	5 (83.3)	7	1	15	0.427	0.829	0.057

Relationship between MA incidence and age, logMAR VA, or AH cytokine levels in nAMD eyes under IVA treatment for 2 years was evaluated by Fisher’s exact test. Abbreviations are as shown in **Tables 1** and **2**. High-value subgroup, nAMD eyes with the cutoff value or more; low-value subgroup, nAMD eyes with less than the cutoff value; *number of nAMD eyes developing MA under IVA for 2 years; ^†^percentage of MA incidence in high-value subgroups; Cramer’s V, Cramer’s coefficient of association; logMAR, logarithm of the minimum angle of resolution; Yule’s Q, Yule’s coefficient of association. Examination items with significant differences and its p values are shown in bold.

The pre-IVA variables of age, logMAR VA, MCP-1, MIP-1β, and VEGF levels were strongly associated with MA incidence in nAMD eyes under IVA treatment for 2 years. Regarding post-IVA variables, logMAR VA and IL-6 level were significantly associated with MA development, although MA incidence was independent of post-IVA MCP-1 and MIP-1β levels.

### Probability of Incidence of Macular Atrophy Depending on Age, Visual Acuity, and Levels of Aqueous Humor Cytokines in nAMD Eyes Under Aflibercept Treatment

**Figure 5:**
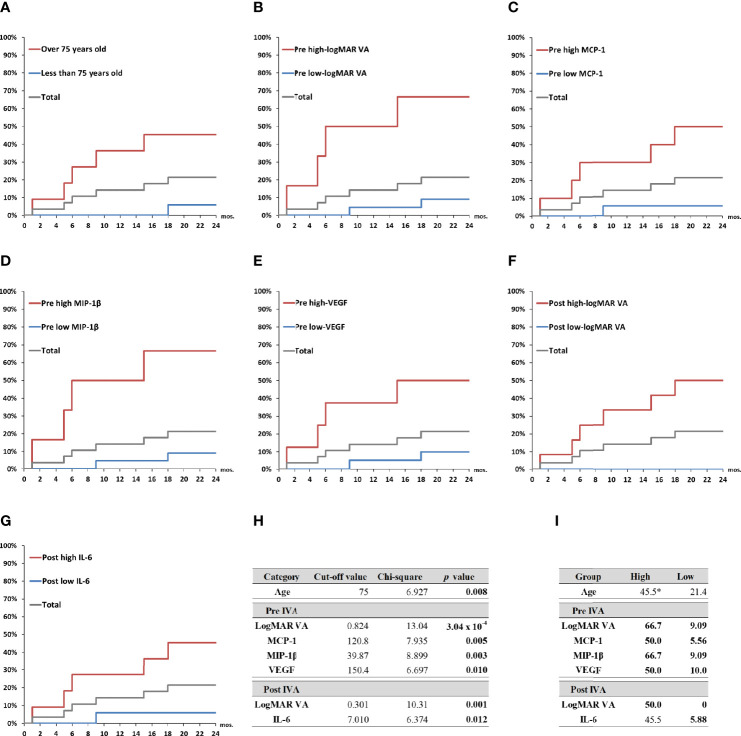
Probability of incidence of macular atrophy depending on age, visual acuity, and aqueous humor cytokine levels in nAMD eyes under aflibercept treatment. Kaplan–Meier survival curves were used to estimate the probability of MA incidence in nAMD eyes under IVA treatment for 2 years. The nAMD group was divided by cutoff values: **(A)** age over or under 75 years at baseline, **(B)** pre-IVA logMAR VA higher or lower than 0.824, **(C)** pre-IVA level of MCP-1 higher or lower than 120.8 pg/ml, **(D)** pre-IVA level of MIP-1β higher or lower than 39.9 pg/ml, **(E)** pre-IVA level of VEGF higher or lower than 150.4 pg/ml, **(F)** post-IVA logMAR VA higher or lower than 0.301, and **(G)** post-IVA level of IL-6 higher or lower than 7.01 pg/ml. **(H)** Values of chi-square and p between high-value and low-value groups divided by each cutoff value. **(I)** Rates of MA incidence in high-value and low-value groups divided by each cutoff value. *percentage; IL, interleukin; IVA, intravitreal injection of aflibercept; MA, macular atrophy; logMAR, logarithm of the minimum angle of resolution; MCP-1, monocyte chemotactic protein-1; MIP-1β, macrophage inflammatory protein; mon., month; nAMD, neovascular age-related macular degeneration; VA, visual acuity; VEGF, vascular endothelial growth factor.

The predictive factors detected by the ROC curve analysis and confirmed by the Fisher’s exact test were adopted as explanatory variables in Kaplan–Meier survival curve analysis (**Figure 5**). Total nAMD group was divided into two subgroups as follows: high-value subgroup and low-value subgroup. For all the predictive factors (age; pre-IVA logMAR VA, MCP-1, MIP-1β, and VEGF; and post-IVA logMAR VA and IL-6), the 2-year MA incidence rates were significantly higher in high-value subgroups than those in low-value subgroups. Especially for the variables of pre-IVA logMAR VA, MCP-1, MIP-1β, and VEGF and post-IVA logMAR VA, the MA incidence rates were over 50% in high-value subgroups and 10% or lower in low-value subgroups (**Figure 5I**). These five parameters were further analyzed as shown below.

### Two-Year Clinical Courses of Visual Acuity and Central Macula Thickness in nAMD Groups Divided by Macular Atrophy Status, Visual Acuity, or Aqueous Humor Cytokine Levels

**Figure 6:**
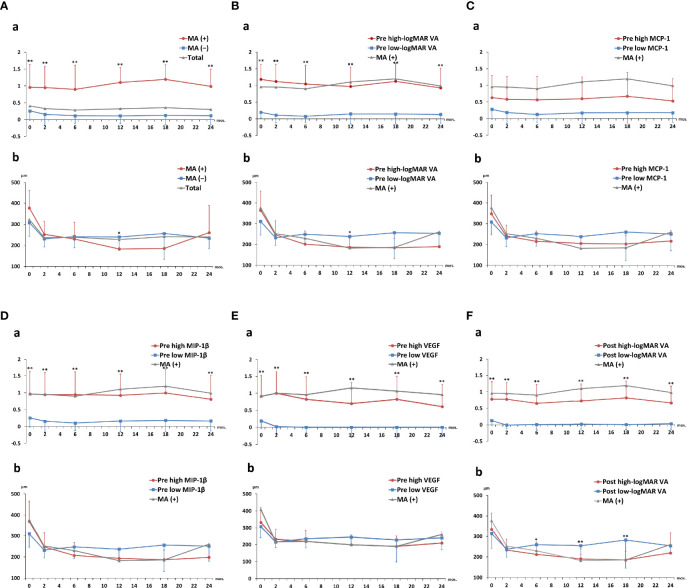
Two-year clinical courses of visual acuity and central macula thickness in nAMD group divided by the presence of macular atrophy, visual acuity, or levels of aqueous humor cytokines. Two-year clinical courses of **(a)** logMAR VA and **(b)** CRT in nAMD eyes under IVA treatment for 2 years, classified by **(A)** presence or absence of MA incidence and above or below cutoff values of **(B)** pre-IVA logMAR VA, **(C)** pre-IVA level of MCP-1, **(D)** pre-IVA level of MIP-1β, **(E)** pre-IVA level of VEGF, and **(F)** post-IVA logMAR VA are shown. The cutoff values were the same as those used in Kaplan–Meier curves (). Data are expressed in mean (close circle) and standard deviation (error bar). CRT, central retinal thickness; IVA, intravitreal injection of aflibercept; MA, macular atrophy; logMAR, logarithm of the minimum angle of resolution; MCP-1, monocyte chemotactic protein-1; MIP-1β, macrophage inflammatory protein; nAMD, neovascular age-related macular degeneration; VA, visual acuity; VEGF, vascular endothelial growth factor.

**Figure 6** shows the 2-year time courses of logMAR VA and CRT in total nAMD, MA (+) and MA (−) groups, as well as in the high-value and low-value subgroups of pre-IVA logMAR VA, MCP-1, MIP-1β, VEGF, and post-IVA logMAR VA. LogMAR VAs were significantly higher in MA (+) group than those in MA (−) group at all the time points throughout the 2-year period (**Figure 6A-a**), although CRTs were not significantly different between MA (+) and MA (−) groups in the course of 2 years except only at 12 months (**Figure 6A-b**). Next, when the subjects were classified by the cutoff value of pre-IVA logMAR VA, logMAR VAs were significantly higher in the high-value subgroup than those in the low-value subgroup at all time points throughout the 2-year period (**Figure 6B-a**), although CRTs were not significantly different between the two subgroups except only at 12 months (**Figure 6B-b**). Besides, when classified by the cutoff value of post-IVA logMAR VA, logMAR VAs were also higher in the high-value subgroup than those in the low-value subgroup at all time points (**Figure 6F-a**), while CRTs were lower in the high-value subgroup than those in the low-value subgroup at 6, 12, and 18 months (**Figure 6F-b**).

Regarding the subgroups classified by the cutoff levels of cytokines, there was no significant difference in logMAR VA and CRT between the high-level and low-level subgroups of pre-IVA MCP-1 at all the time points (**Figure 6C**). On the other hand, logMAR VAs were higher in the high-level subgroups of pre-IVA MIP-1β and VEGF compared to the low-level subgroups of these cytokines at all time points (**Figures 6D-a, E-a**, respectively), although there was no significant difference in CRT between the high- and low-level subgroups of these cytokines. In the subgroups classified by the cutoff values of age and post-IVA IL-6 level, logMAR VA was significantly higher at age over 75 years than that at age under 75 years only at 12 months (**Supplementary Figure S3A-a**), although CRT was not significantly different between the two subgroups. There was no significant difference in logMAR VA and CRT between the high- and low-level subgroups of post-IVA IL-6 at all time points (**Supplementary Figure S3B**). The p-values obtained by comparing logMAR VA and CRT between the high- and low-value subgroups at various time points during 2 years of IVA treatment are shown in **Supplementary Table S5**.

Multiple comparisons of logMAR VA and CRT over the course of 2 years were also performed in nAMD eyes divided into MA (+) group and MA (−) group (**Supplementary Table S6**), as well as by the cutoff values of pre-IVA logMAR VA (**Supplementary Table S7**), pre-IVA MCP-1 (**Supplementary Table S8**), pre-IVA MIP-1β (**Supplementary Table S9**), pre-IVA VEGF (**Supplementary Table S10**), post-IVA logMAR VA (**Supplementary Table S11**), age (**Supplementary Table S12**), and post-IVA IL-6 (**Supplementary Table S13**).

## Discussion

The present study examined MA-associated factors and identified significant biomarkers that predict MA incidence in nAMD eyes under IVA treatment for 2 years. Several major findings involved in MA incidence were obtained as follows: 1) Hierarchical cluster analysis and PCA show that IP-10 is closely associated with CRT, an indicator of MA pathology before IVA treatment, but this association was lost after initiation of IVA treatment; 2) The associations of MCP-1 and MIP-1β with MA pathology did not change remarkably before and after initiation of IVA treatment; 3) After initiation of IVA, MCP-1 correlated positively with CRT, and MIP-1β level correlated positively with logMAR VA; and 4) Pre-IVA MIP-1β and VEGF levels were identified as significant biomarkers for predicting the incidence of MA in nAMD eyes under IVA treatment.

Ocular fluids comprising AH and vitreous humor have been reported to reflect intraocular immune environment in AMD (17, 18, 31), proliferative diabetic retinopathy (32), and uveitis (33, 34). Since sampling of AH is easier and safer compared to vitreous fluid (17, 18), AH has been recognized as an ideal biological specimen to examine the pathology of ocular diseases (35). Our group has reported the participation of inflammatory cytokines including IP-10, MCP-1, and MIP-1β in the pathophysiology of nAMD (17, 18).

Recently, much attention has been focused on the incidence of MA in nAMD eyes under anti-VEGF therapy as a serious adverse effect that exacerbates VA and central visual field (11). Large-scale clinical studies have examined the associations of dose (11), treatment regimen (11–13), and types of anti-VEGF antibodies (14, 36) with the development of MA in nAMD eyes under anti-VEGF therapy. However, the etiology of MA remains unclear, and there is no effective treatment for this condition (11). In this study, the specific expression patterns of AH cytokines associated with the development of MA were examined, aiming to help understand the immune mechanisms of MA.

In this study, hierarchical cluster analysis shows that before initiation of IVA, IP-10 and CRT form a principal cluster, showing close association of IP-10 with nAMD. After initiation of IVA, however, IP-10 clusters independently from CRT, suggesting that the pathological significance of IP-10 in MA is lost (**Figure 2**). This finding is supported by the results of PCA for MA (+) group, which suggest that IP-10 contributes to some extent to the pathology of MA before IVA initiation, but the contribution is diminished after IVA initiation (**Figure 3**). IP-10 is a chemokine that attracts type 1 T helper (Th1) cells and activates Th1 cell-mediated immune responses (37), which are responsible for the development of nAMD (38, 39). In terms of cytokine action, IP-10 works as an antiangiogenic and antifibrotic substance (40, 41). On the other hand, the pre-IVA level of VEGF was higher in MA (+) group than that in MA (−) group. Elevated VEGF level is a significant feature of nAMD eyes with high susceptibility of developing MA because the angiogenic effect of VEGF could be assumed to compete with the antiangiogenic effect of IP-10 before IVA initiation, resulting in the suppression of fibrosis and atrophy in the injured macula. Sadda et al. (11) proposed the potential mechanisms of pathology in MA as follows: 1) natural progression of underlying dry AMD; 2) collateral impact of the extension/retraction of CNV; and 3) interference with basal VEGF levels. Therefore, we hypothesize another potential mechanism of MA development in nAMD eyes under IVA treatment as follows: the antifibrotic immune reactions mediated by IP-10 become insufficient when intraocular VEGF is depleted following aflibercept treatment, and as a result, fibrosis and atrophy would progress under this altered condition.

In the present study, the associations and contributions of some inflammatory cytokines to the pathology of nAMD changed remarkably after initiation of aflibercept treatment that almost depleted intraocular VEGF. Hierarchical cluster analysis indicated that while MCP-1 and VEGF were grouped closely together before IVA initiation, MCP-1 was grouped with CRT after IVA initiation in MA (+) group (**Figure 2**). However, the pathological contribution of MCP-1 was significant before IVA initiation, and this situation continued after the initiation (**Figure 3**). Furthermore, post-IVA MCP- 1 level correlated positively with post-IVA CRT in MA (+) group (**Table 3**). MCP-1 is one of the key chemokines regulating migration and infiltration of monocytes/macrophages (42). Previous studies using AH or plasma reported the participation of MCP-1 in the pathophysiology of nAMD (5, 19, 43). Jonas et al. (19) reported a positive correlation between macula thickness and AH level of MCP-1 in treatment-naive nAMD eyes. On the other hand, Müller glial cells perform key housekeeping, osmoregulatory, and mechanosensory functions in the retina (44). In an *in vivo* model of non-rhegmatogenous retinal detachment (RRD) with SRF, non-RRD induced swelling of Müller glial cells, and the swelling caused MCP-1 release from the cells, resulting in recruitment of macrophages that attack and kill photoreceptors (45). Therefore, we speculate that MCP-1 is a cytokine responsible for CRT in nAMD eyes through inducing osmotic imbalance and chronic inflammation. More research is needed to clarify the potential functions of MCP-1 in the pathology of nAMD.

In MA (+) group, the post-IVA MIP-1β level correlated positively with post-IVA logMAR VA (**Table 3**), whereas there was no correlation between the pre- and post-IVA levels of MIP-1β (**Supplementary Figure S1**), implying that high MIP-1β level was not an individual patient’s characteristic but a feature of the MA (+) group. MIP-1β is a member of the CC chemokine family that recruits macrophages/microglia to the injury sites in patients with arthritis (46), sepsis (47), and systemic sclerosis (48). In mouse retina, MIP-1β level was elevated rapidly under hypoxic stimulation (49). Coleman et al. (50) reported that ischemia and inflammation in the choroid were pathological factors for developing dry AMD. Besides, the local inflammation was activated in the RPE–Bruch’s membrane–choriocapillaris complex of AMD eyes (51). Therefore, it is possible that MIP-1β is an MA-associated factor in nAMD through inducing ischemia and chronic inflammation in the choroid.

Molecular diagnostic tests drive scientific and technological progress in the fields of predictive, preventive, and personalized medicine and facilitate early detection, monitoring, and risk assessment of diseases as well as guide therapeutic decision (52). Furthermore, the results of diagnostic tests have immense impact, affecting around 60%–70% of all clinical decisions, although they still amount for only 4%–5% in healthcare costs (53). Thus, diagnostic tests with high accuracy using minimally invasive sampling specimens are useful to improve quality of life and to avoid economic burden for both the individuals and society.

In the present study, age and pre-IVA logMAR VA, pre-IVA levels of MCP-1, MIP-1β, and VEGF, as well as post-IVA logMAR VA and post-IVA levels of IL-6, MCP-1, and MIP-1β were detected as significant factors for predicting the incidence of MA by ROC curve analysis and Kaplan–Meier survival curve. The clinical courses of VA and CRT in the high-value subgroups of pre-IVA logMAR VA, MIP-1β, and VEGF and high-value subgroup of post-IVA logMAR VA were quite similar to those in MA (+) group. Therefore, pre-IVA MIP-1β and VEGF levels were selected as significant biomarkers for predicting the incidence of MA. The incomplete match of the predictive biomarkers obtained by ROC curve analysis and the clinical courses may be attributed to the following reasons: The clinical characteristics of individual cytokines may be distinct before IVA treatment but may change to resemble each other after initiation of IVA (**Figure 3**). In other words, some unique functions of individual cytokines might be lost when intraocular VEGF is depleted in nAMD eyes susceptible to MA development.

Fundus images derived from OCT as central subfield thickness (CST) are clinically used to inform retreatment decisions of anti-VEGF therapies in nAMD eyes (23, 54). CST at baseline in nAMD eyes usually had a negative correlation with VA, but the correlation was less evident during follow-up (55, 56). In *post-hoc* analyses of clinical trials (57–60) and real-world study (61), certain baseline morphological parameters including IRF, SRF, subretinal hyperreflective material (SHRM), and pigment epithelium detachment (PED) were associated with visual outcomes in nAMD eyes beginning anti-VEGF therapy. Therefore, IRF, SRF, SHRM, and PED have been recognized as significant indicators of disease activity in macular neovascularization (62), and the elevations of IRF and age are expected as negative prognostic impact on visual outcomes (57, 58, 63, 64). As for the development of MA in nAMD eyes under anti-VEGF therapy, IRF in foveal center at baseline was identified as a risk factor for MA incidence in the Comparison of Age-Related Macular Degeneration Treatments Trials (12). Furthermore, lower volumes of both the RPE and the neurosensory retina might be observed in nAMD cases with MA (65). In the future, further study of examining OCT-derived morphological parameters combined with AH cytokine is expected to reveal the pathophysiology of AMD that may help optimize disease management and offer the possibility of more personalized medicine.

At present, machine learning, which is a subset of artificial intelligence, has been used as a data-driven analytic approach for the prediction of onset and progression of various diseases (66). In ophthalmology, several machine learning methods have been developed to classify diabetic retinopathy, AMD, retinopathy of prematurity, and glaucoma based on fundus images and OCT images (67–71). Nezu et al. (66) recently reported that random forest algorithms using AH cytokines predicted the diagnosis of vitreoretinal lymphoma, acute retinal necrosis, and endophthalmitis. In the future, machine learning combined with AH cytokines would be a promising diagnosis method and contribute to find new biomarkers that could be an aid to diagnose ocular diseases.

The present study has several limitations. 1, The number of enrolled nAMD patients was not large enough to perform subgroup analysis for each phenotype of nAMD. 2, nAMD eyes under aflibercept treatment were only examined, although other anti-VEGF antibodies including pegaptanib (6), ranibizumab (7), and brolucizumab (9) as well as bevacizumab (10) have been used in the treatments for nAMD. 3, All the subjects were Japanese, although the proportion of PCV is markedly higher in Asian compared to Caucasian (72, 73). 4, The thickness of all layers in the macula was only examined as an OCT-derived morphological parameter, although subtypes of the parameters as IRF, SRF, PED, and SHRM (12, 68, 74–76) were not evaluated. 5, The *PRN* regimen was used in this study, while other regimens (11–13) such as fixed-dose and treat-and-extend were not examined. 6, The items evaluated were limited to 27 inflammatory cytokines, VA, and CRT.

## Conclusions

In conclusion, various inflammatory reactions interact with each other in a complicated manner in the pathology of nAMD. In nAMD eyes susceptible to the development of MA under IVA treatment, IP-10 as a VEGF antagonist and antifibrotic agent is closely associated with CRT before IVA treatment. After initiation of IVA treatment, however, the protective effects of IP-10 on nAMD are lost under depletion of intraocular VEGF, resulting in progression of atrophy with chronic inflammation induced by MCP-1 and MIP-1β. Furthermore, AH cytokine levels before IVA initiation, especially MIP-1β and VEGF levels, are significant biomarkers for predicting the incidence of MA in nAMD eyes under IVA treatment. In the future, new molecular targeted therapy as an adjuvant treatment is expected to be useful for nAMD patients with poor response to anti-VEGF therapy.

## Data Availability Statement

The original contributions presented in the study are included in the article/**Supplementary Material**. Further inquiries can be directed to the corresponding author.

## Ethics Statement

The studies involving human participants were reviewed and approved by the National Defense Medical College Hospital and Enoki Eye Clinic in Japan. The patients/participants provided their written informed consent to participate in this study.

## Author Contributions

TS and MTak designed the study. T. Sato, TS, TE, and MTak performed experiments. TS, TE, YK, KT, and MTak collected clinical information and classified patients. TS, HS, MTag, KH, TK, KT,MI, and MTak performed statistical analysis and drafted the manuscript. All authors contributed to the article and approved the submitted version.

## Funding

This study was supported by Grant-in-Aid for Scientific Research C from the Japan Society for the Promotion of Science (16K11337), Grant-in-Aid for Encouragement of Young doctors from National Defense Medical College, Research Grant from Daiwa Securities Health Foundation, Hisakichi Matsubayashi Memorial Fund Subsidy, and Grant-in-Aid for Advanced Medical Development from National Defense Medical College. This study received funding from Novartis Research Grant and Alcon Research Grant. The funders were not involved in the study design, collection, analysis, interpretation of data, the writing of this article, or the decision to submit it for publication.

## Conflict of Interest

The authors declare that the research was conducted in the absence of any commercial or financial relationships that could be construed as a potential conflict of interest.

## Publisher’s Note

All claims expressed in this article are solely those of the authors and do not necessarily represent those of their affiliated organizations, or those of the publisher, the editors and the reviewers. Any product that may be evaluated in this article, or claim that may be made by its manufacturer, is not guaranteed or endorsed by the publisher.

## Abbreviations

AH, aqueous humor; AMD, age-related macular degeneration; BCVA, best-corrected visual acuity; bFGF, basic fibroblast growth factor; CCR, cumulative contribution ratio; CNV, choroidal neovascularization; CR, contribution ratio; Cramer’s V, Cramer’s coefficient of association; CST, central subfield thickness; CRT, central retinal thickness; F, female; G-CSF, granulocyte colony-stimulating factor; GM-CSF, granulocyte-macrophage colony-stimulating factor; IFN-γ, interferon-gamma; IL, interleukin; IOL, intraocular lens; IP-10, interferon γ-inducible protein 10; IRF, intraretinal fluid; IVA, intravitreal injection of aflibercept; LPI, laser peripheral iridotomy; M, male; MA, macular atrophy; MAR, minimum angle of resolution; MA (+) group, nAMD eyes developing MA; MA (−) group, nAMD eyes without MA; MCP-1, monocyte chemotactic protein-1; MIP, macrophage inflammatory protein; mos., months; *n*, number; nAMD, neovascular AMD; PC, principal component; PCA, principal component analysis; PCL, principal component loading; PCV, polypoidal choroidal vasculopathy; PDGF-BB, platelet-derived growth factor-BB; PED, pigment epithelium detachment; post-IVA, before the third IVA; pre-IVA, before first IVA; PC1, first principal component; PC2, second principal component; ra, receptor antagonist; RANTES, regulated on activation, normal T cell expressed and secreted; RAP, retinal angiomatous proliferation; RPE, retinal pigment epithelium; ROC, receiver operating characteristic; RRD, rhegmatogenous retinal detachment; SD, standard deviation; SD-OCT, spectral-domain optical coherence tomography; SHRM, subretinal hyperreflective material; SRF, subretinal fluid; Th1, type 1 T helper; TNFα, tumor necrosis factor α; VA, visual acuity; VEGF, vascular endothelial growth factor; Yule’s Q, Yule’s coefficient of association.
