# Supplementary material for: Inflammatory Factors of Macular Atrophy in Eyes With Neovascular Age-Related Macular Degeneration Treated With Aflibercept
Source: Front Immunol. 2021 Oct 13;12:738521. doi: 10.3389/fimmu.2021.738521 (PMC8548619; doi:10.3389/fimmu.2021.738521)
Supplement: Supplementary file 1 [file DataSheet_1.docx]

**Supplemental Figure 1. Correlation between pre- and post-IVA values of visual acuity, central macula thickness or aqueous humor cytokines in nAMD group developing macula atrophy under aflibercept treatment for 2 years.**

**
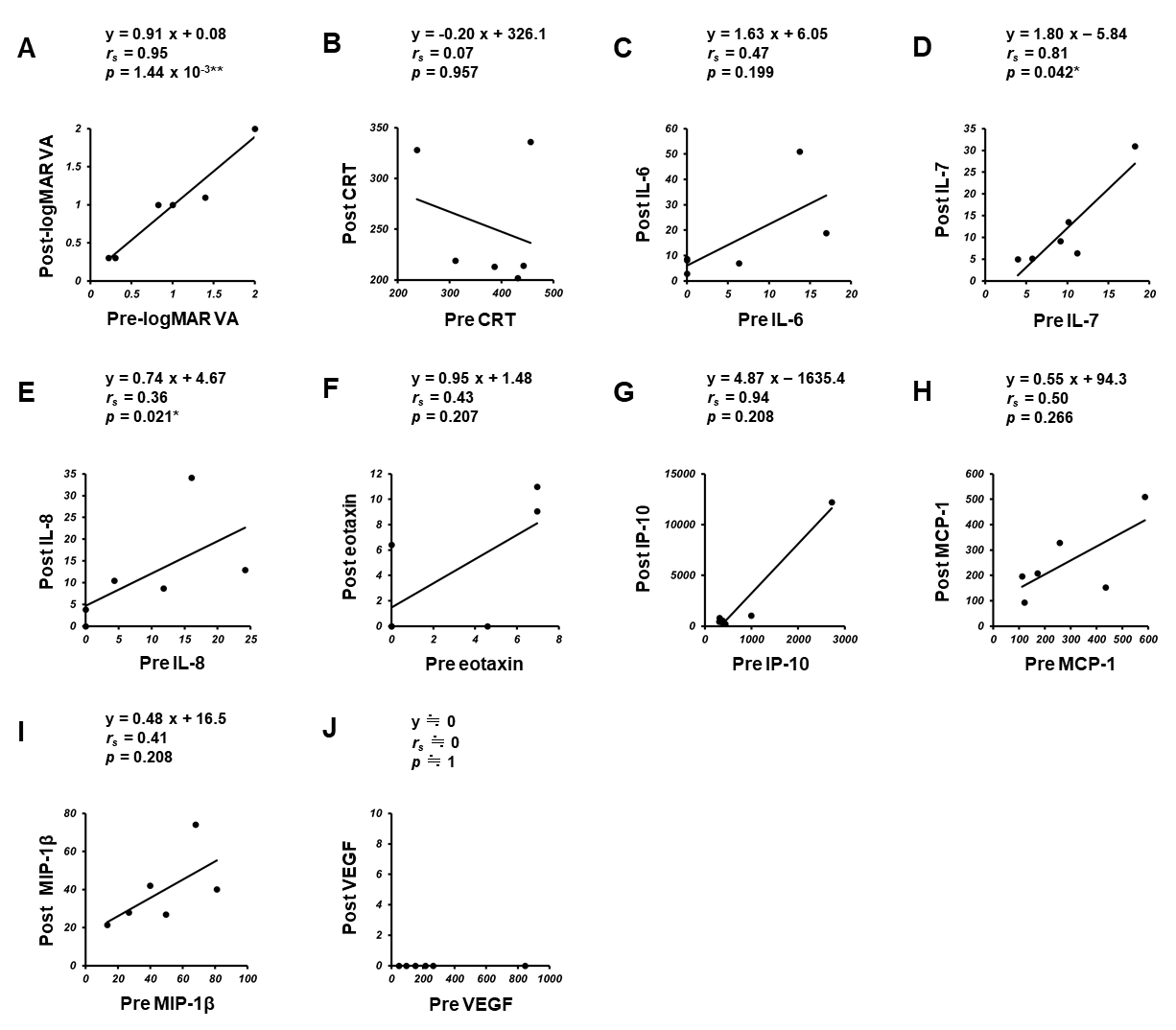
**

CRT is expressed in μm, and cytokine levels in pg/mL. IL; interleukin, IP-10; interferon gamma-inducible protein 10, IVA; intravitreal injection of aflibercept, MAR; minimum angular resolution, MCP-1; monocyte chemotactic protein-1, MIP; macrophage inflammatory protein, nAMD; neovascular age-related macular degeneration, post; before third intravitreal injection of aflibercept, pre; before first intravitreal injection of aflibercept, *r_s_*; Spearman correlation coefficient, VA; visual acuity, VEGF; vascular endothelial growth factor, *; *p* < 0.05, **; *p* < 0.01, ≒; nearly equal.

**Supplemental Figure 2. Correlation between pre- and post-IVA values of visual acuity, central macula thickness or aqueous humor cytokines in nAMD group without macula atrophy under aflibercept treatment for 2 years.**

**
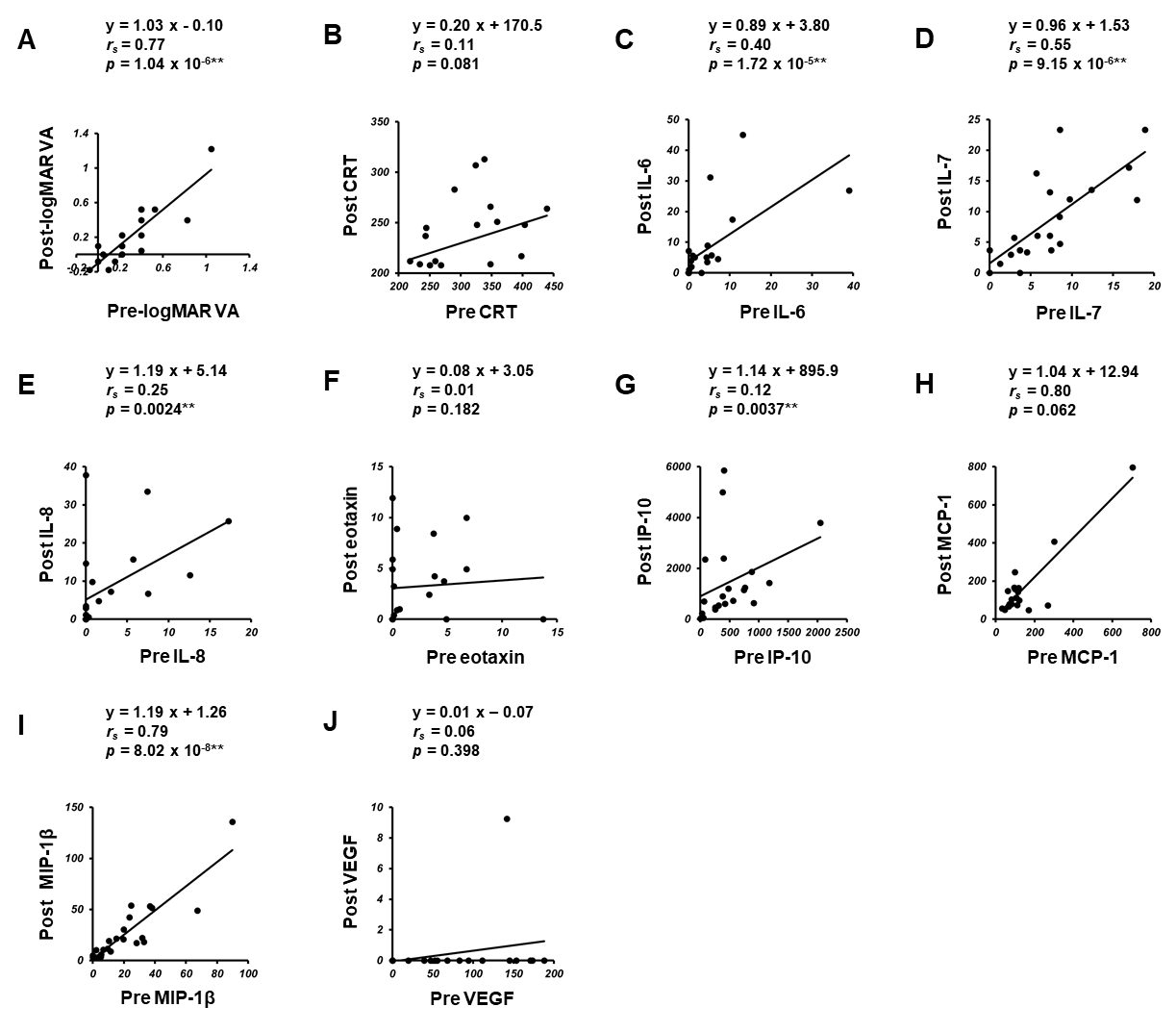
**

**Supplemental Figure 3. Two-year time courses of visual acuity and central macula thickness in nAMD groups divided by age or aqueous humor level of IL-6 after aflibercept treatment.**

**
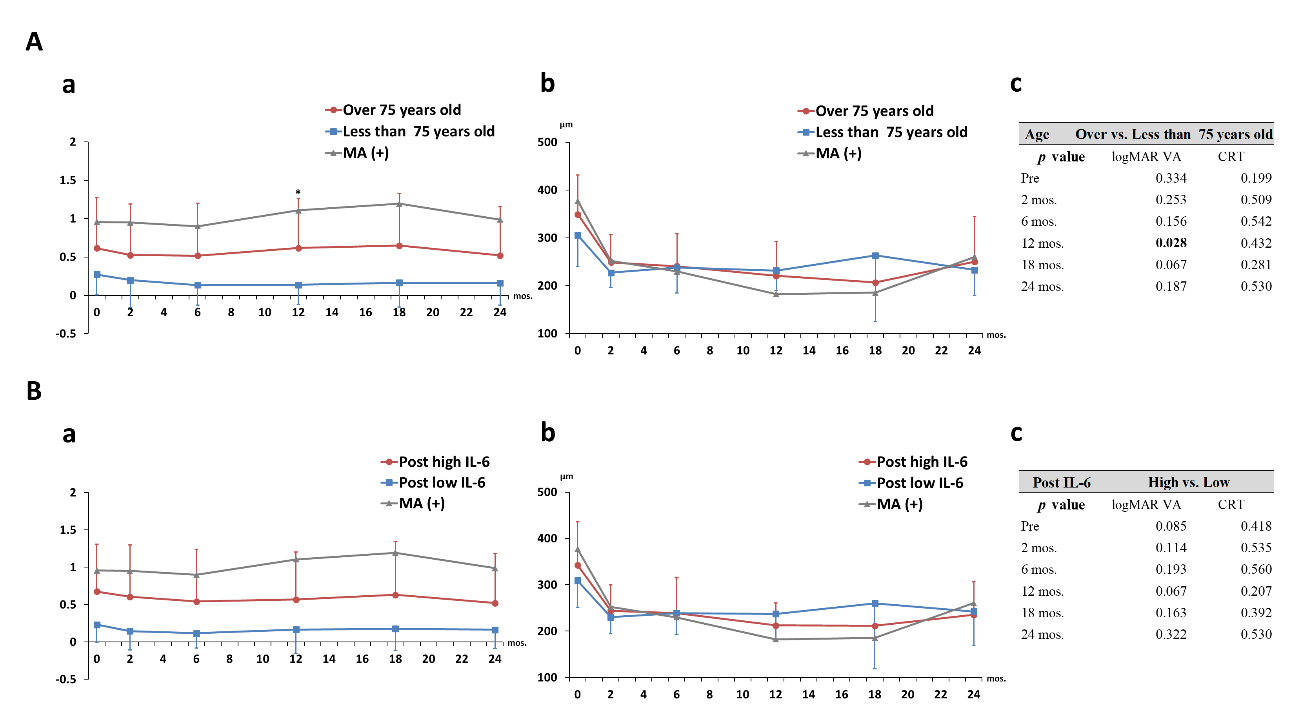
**

Clinical courses and comparisons of (**a**) logMAR VA and (**b**) CRT in nAMD groups under IVA for 2 years divided by (**A**) age above or under 75 years, (**B**) post-IVA level of IL-6 higher or lower than 7.01 pg/mL. (**c**) *P* values between high- and low-value groups divided by each cutoff value. Data is expressed in mean (closed circle) and standard deviation (error bar). MA; macula atrophy, MA (+); nAMD eyes developing MA, mos.; months.
