# Supplementary material for: Inflammatory Factors of Macular Atrophy in Eyes With Neovascular Age-Related Macular Degeneration Treated With Aflibercept
Source: Front Immunol. 2021 Oct 13;12:738521. doi: 10.3389/fimmu.2021.738521 (PMC8548619; doi:10.3389/fimmu.2021.738521)
Supplement: Supplementary file 2 [file DataSheet_2.docx]

**Supplemental Table 1. Clinical characteristics and number of aflibercept treatment for 2 years in nAMD patients and controls.**

(**A**) Clinical characteristics in nAMD patients and controls, (**B**) number of IVA doses for 2 years, in nAMD patients are shown. F; female, M; male, mo.; month, *n*; number, nAMD; neovascular age-related macular degeneration, PCV; polypoidal choroidal vasculopathy, RAP; retinal angiomatous proliferation, SD; standard deviation, ^#^; average, ^†^; range.

**Supplemental Table 2.** **Visual acuity, central retinal thickness and aqueous humor cytokine levels in nAMD patients and controls.**

LogMAR VA, CRT and AH cytokine levels in nAMD patients and controls are shown. CRT is expressed in μm, and cytokine levels in pg/mL. AH; aqueous humor, bFGF; basic-fibroblast growth factor, CRT; central retinal thickness, G-CSF; granulocyte colony-stimulating factor, GM-CSF; granulocyte-macrophage colony-stimulating factor, PDGF-BB; platelet-derived growth factor-BB, IL; interleukin, IFN-γ; interferon-gamma, IP-10; interferon gamma-inducible protein 10, IVA; intravitreal injection of aflibercept, pre-IVA; before first IVA, post-IVA; before the third IVA, MAR; minimum angular resolution, MCP-1; monocyte chemotactic protein-1, MIP; macrophage inflammatory protein, ra; receptor antagonist, RANTES; regulated on activation, normal T cell expressed and secreted, TNFα; tumor necrosis factor α, VA; visual acuity, VEGF; vascular endothelial growth factor.

**Supplemental Table 3. Spearman's rank correlation coefficients for correlation between aqueous humor cytokine levels and visual acuity or central macula thickness in nAMD eyes with or without incidence of macula atrophy under aflibercept treatment.**

Results of *r_s_* in Spearman correlation between levels of aqueous humor cytokines and logMAR VA or CRT are described. MA; macula atrophy, MA (+); nAMD eyes developing MA, MA (−); nAMD eyes without MA, *r_s_*; spearman correlation coefficient, *; moderate positive correlation (*r_s_*, 0.4 ≥ and < 0.70), **; high positive correlation (*r_s_*, 0.70 ≥).

**Supplemental Table 4. Parameter values of factors for predicting incidence of macula atrophy by receiver operating characteristic curves in nAMD eyes under aflibercept treatment.**

Predictive factors for MA incidence in nAMD eyes under aflibercept treatment were composed of age (years), logMAR VA and cytokines with over 50% detection rate. Cut-off value is defined by closest point to upper left-hand corner of graph in receiver operating characteristic curve. AUC; area under the curve, NPV; negative predictive value, OR; odds ratio, PPV; positive predictive value, SE; standard error.

**Supplemental Table 5. Comparisons of visual acuity and central macula thickness between nAMD eyes classified by presence or absence of macula atrophy and by cut-off values of visual acuity and aqueous humor cytokine levels.**

Comparisons of logMAR VA and CRT at the indicated periods during 2 years of aflibercept treatment in nAMD eyes classified by (**A**) presence or absence of MA incidence, and higher or lower than cut-off value of (**B**) pre-IVA logMAR VA, (**C**) pre-IVA MCP-1 level, (**D**) pre-IVA MIP-1β level, (**E**) pre-IVA VEGF level or (**F**) post-IVA logMAR VA are shown. mos.; months.

**Supplemental Table 6. Multiple comparisons of visual acuity and central macula thickness in nAMD eyes under aflibercept treatment for 2 years, divided by presence or absence of MA incidence.**

Multiple comparisons of logMAR VA and CRT at the indicated periods in nAMD eyes under aflibercept treatment for 2 years, divided by presence or absence of MA incidence are shown. Total; group of all nAMD eyes enrolled. *; *p* < 0.05, **; *p* < 0.01.

**Supplemental Table 7. Multiple comparisons of visual acuity and central macula thickness in nAMD eyes divided by cut-off value of pre-IVA logMAR VA.**


Multiple comparisons of logMAR VA and CRT in nAMD eyes under aflibercept treatment for 2 years, divided by pre-IVA logMAR VA higher or lower than 0.824 are shown.

**Supplemental Table 8. Multiple comparisons of visual acuity and central macula thickness in nAMD eyes divided by cut-off value of pre-IVA MCP-1 level.**

Multiple comparisons of logMAR VA and CRT in nAMD eyes under aflibercept treatment for 2 years divided by pre-IVA MCP-1 level higher or lower than 120.8 pg/mL are shown.

**Supplemental Table 9. Multiple comparisons of visual acuity and central macula thickness in nAMD eyes divided by cut-off value of pre-IVA MIP-1β level.**

Multiple comparisons of logMAR VA and CRT in nAMD eyes under aflibercept treatment for 2 years divided by pre-IVA MIP-1β level higher or lower than 39.9 pg/mL are shown.

**Supplemental Table 10. Multiple comparisons of visual acuity and central macula thickness in nAMD eyes divided by cut-off value of pre-IVA VEGF level.**

Multiple comparisons of logMAR VA and CRT in nAMD eyes under aflibercept treatment for 2 years divided by pre-IVA VEGF level higher or lower than 150.4 pg/mL are shown.

**Supplemental Table 11. Multiple comparisons of visual acuity and central macula thickness in nAMD eyes divided by cut-off value of post-IVA logMAR VA.**

Multiple comparisons of logMAR VA and CRT in nAMD eyes under aflibercept treatment for 2 years divided by post-IVA logMAR VA higher or lower than 0.301 are shown.

**Supplemental Table 12. Multiple comparisons of visual acuity and central macula thickness in nAMD eyes divided by cut-off value of age.**

Multiple comparisons of logMAR VA and CRT in nAMD eyes under aflibercept treatment for 2 years divided by age over or under 75 years at baseline are shown.

**Supplemental Table 13. Multiple comparisons of visual acuity and central macula thickness in nAMD eyes divided by cut-off value of post-IVA IL-6 level.**

Multiple comparisons of logMAR VA and CRT in nAMD eyes under aflibercept treatment for 2 years divided by post-IVA IL-6 level higher or lower than 7.01 pg/mL are shown.
